# [Retracted] PPM1A as a key target of the application of Jiawei-Maxing-Shigan decoction for the attenuation of radiation-induced epithelial-mesenchymal transition in type II alveolar epithelial cells

**DOI:** 10.3892/mmr.2026.13971

**Published:** 2026-07-27

**Authors:** Jinhua Lu, Shengyou Lin, Zechen Lin, Xianlei Lin, Yuezhong Shen, Jingyang Su

Mol Med Rep 24: 825, 2021; DOI: 10.3892/mmr.2021.12465

Following the publication of the above paper, it was drawn to the Editor's attention by a concerned reader that certain of the western blot data featured in Figs. 2B, 4B, 5C and 6A had either already been submitted for publication, or were already published, in several articles in different journals written by different authors at different research institutes. A subsequent investigation of this paper underaken by the Editorial Office confirmed the authenticity of these findings.

Owing to the fact that the contentious data in the above article had either already been submitted for publication, or were already published, prior to its submission to *Molecular Medicine Reports*, the Editor has decided that this paper should be retracted from the Journal. The authors were asked for an explanation to account for these concerns, but the Editorial Office did not receive a reply. The Editor apologizes to the readership for any inconvenience caused.

